# Combinatorial Regimen of Carbamazepine and Imipramine Exhibits Synergism against Grandmal Epilepsy in Rats: Inhibition of Pro-Inflammatory Cytokines and PI3K/Akt/mTOR Signaling Pathway

**DOI:** 10.3390/ph14111204

**Published:** 2021-11-22

**Authors:** Faheem Hyder Pottoo, Mohammed Salahuddin, Firdos Alam Khan, Marwa Abdullah AL Dhamen, Walaa Jafar Alsaeed, Mohamed S. Gomaa, Chittibabu Vatte, Mohammad N. Alomary

**Affiliations:** 1 Department of Pharmacology, College of Clinical Pharmacy, Imam Abdul Rahman Bin Faisal University, P.O. Box 1982, Dammam 31441, Saudi Arabia; 2160005543@iau.edu.sa (M.A.A.D.); 2160000663@iau.edu.sa (W.J.A.); 2 Department of Clinical Pharmacy Research, Institute for Research and Medical Consultation, Imam Abdul Rahman Bin Faisal University, P.O. Box 1982, Dammam 31441, Saudi Arabia; msalahuddin@iau.edu.sa; 3 Department of Stem cell Research, Institute for Research and Medical Consultation, Imam Abdul Rahman Bin Faisal University, P.O. Box 1982, Dammam 31441, Saudi Arabia; fakhan@iau.edu.sa; 4 Department of Pharmaceutical Chemistry, College of Clinical Pharmacy, Imam Abdul Rahman Bin Faisal University, P.O. Box 1982, Dammam 31441, Saudi Arabia; msmmansour@iau.edu.sa; 5 Department of Biochemistry, College of Medicine, Imam Abdul Rahman Bin Faisal University, P.O. Box 1982, Dammam 31441, Saudi Arabia; cbvatte@iau.edu.sa; 6 National Centre for Biotechnology, Kind Abdulaziz City for Science and Technology (KACST), P.O. Box 1982, Riyadh 11442, Saudi Arabia

**Keywords:** epilepsy, neurodegenerative disorder, seizures, PI3K/Akt/mTOR signaling, inflammation, cooperative binding, molecular docking, drug repurposing

## Abstract

Epilepsy is a neurodegenerative disorder that causes recurring seizures. Thirty-five percent of patients remain refractory, with a higher prevalence of depression. We investigated the anticonvulsant efficacy of carbamazepine (CBZ; 20 and 50 mg/kg), imipramine (IMI; 10 and 20 mg/kg) alone, and as a low dose combination. This preclinical investigation included dosing of rats for 14 days followed by elicitation of electroshock on the last day of treatment. Along with behavioral monitoring, the rat hippocampus was processed for quantification of mTOR, IL-1β, IL-6 and TNF-α levels. The histopathological analysis of rat hippocampus was performed to ascertain neuroprotection. In vitro studies and in silico studies were also conducted. We found that the low dose combinatorial therapy of CBZ (20 mg/kg) + IMI (10 mg/kg) exhibits synergism (*p* < 0.001) in abrogation of maximal electroshock (MES) induced convulsions/tonic hind limb extension (THLE), by reducing levels of pro-inflammatory cytokines, and weakening of the PI3K/Akt/mTOR signal. The combination also exhibits cooperative binding at the Akt. As far as neuroprotection is concerned, the said combination increased cell viability by 166.37% compared to Pentylenetetrazol (PTZ) treated HEK-293 cells. Thus, the combination of CBZ (20 mg/kg) + IMI (10 mg/kg) is a fruitful combination therapy to elevate seizure threshold and provide neuroprotection.

## 1. Introduction

Epilepsy is a chronic neurological disease featuring escalated susceptibility to seizures (hypersynchronous neuronal activity) [1,2,3]. It affects 70 million of the world’s population (90% from developing nations) [4]. In the KSA, the epilepsy prevalence rate is 6.54 per 1000 [5]. The incidence and prevalence of epileptic seizure in males surpass females, and it is more prevalent in the low to middle economic population than the higher economic population. In children and adults, the focal seizures are more common than generalized seizures [6]. Epilepsy leads to neuronal pyknosis in all hippocampal regions [7,8]. Epileptic seizures lead to the production of free radicals and oxidative insult to cellular proteins, lipids and DNA, causing neuronal death [9]. Despite progress in the drug development for all neurodegenerative disorders, including epilepsy, 35% of epileptic patients remain refractory, which badly affects their quality of life (QoL) [10,11]. 

Carbamazepine (CBZ; dibenzoazepine), as an anticonvulsant was first marketed in Europe [12]. It is chemically related to tricyclic antidepressants (TCAs) [11]. Dalby, 1971, reported psychotropic effects (mood stabilization) of CBZ in temporal lobe epilepsy (TLE) patients [13]. It is now indicated in the pharmacotherapy of patients with trigeminal neuralgia, epilepsy, and bipolar I disorder [14]. In epileptic states, CBZ is prescribed for partial seizures, grand mal seizures, and mixed seizure patterns. However, it is not prescribed in the absence seizure type [14]. The mode of action of carbamazepine involves a blockade of voltage-gated Na^+^ channels, which inhibits excessive neuronal firings without interfering with normal non-bursting neuronal transmission [15].

Imipramine (IMI; dibenzazepine-derivative) is a prototype of tricyclic antidepressants (TCAs) [16], structurally related to phenothiazines [17]. IMI reduces the neuronal uptake of norepinephrine (NE) and serotonin (5-HT) by blocking the Na^+^ dependent 5-HT and NE transporters [18], which increases the concentration of NE and 5-HT at the synaptic cleft (thereby modulating the protein kinase signaling and changes in neuro-transmission) and relieving the depressive symptoms [19]. A large number of investigations from rodent and gene knockout studies in mice had revealed the anticonvulsant properties of NE. In addition, the boost of NE levels with the Ketogenic diet manifests as an anticonvulsant effect in rodents [20]. Thus, by increasing the levels of NE in the synaptic cleft, IMI exhibits anticonvulsant effects.

mTOR is a protein from the PI3K-related kinase family having two catalytic subunits of distinct protein complexes, mTOR Complex 1 (mTORC1) and 2 [21]. mTOR regulates the growth and metabolism of eukaryotic cells [22]. mTOR is stimulated by phosphorylation responding to growth factors (such as BDNF), stress and mitogens. The mTOR activity is modulated by various receptors such as dopaminergic, tropomyosin receptor kinase B (TrkB), AMPA and metabotropic glutamate receptors (mGluRs) [23]. Signaling via mTOR is critical for epileptogenic activities [24,25] such as changes in ion channel expression and synaptic plasticity [24,26].

Neuroinflammation is related to the pathophysiology of CNS disorders; depression, Parkinson’s disease (PD), cognitive issues, Alzheimer’s disease (AD), and epilepsy [27,28]. Regarding inflammatory markers, cytokines have an essential role in neurodegenerative processes [27,28]. Some cytokines have an essential function in CNS pathophysiology related to seizures (e.g., IL-1β, IL-6, TNF-α) [27,28]. IL-8 and IL-1β being pro-inflammatory cytokines, escalate seizure vulnerability and organ impairment, while IL-10 receptor agonists are anti-inflammatory cytokines, which have anti-seizure and neuroprotective effects [27,28]. Currently, studies have documented changes in IL-1β levels in CSF, blood and brain tissues [27,28,29,30]. IL-1β levels are higher in generalized tonic-clonic seizure (GTCS) patients than normal patients. Another inflammatory marker of interest is Interleukin 6 (IL-6), primarily a pro-inflammatory cytokine [27,28,29]. After seizure episodes there is an elevated level of IL-6 in the peripheral blood and CSF [27,28,29]. After GTCS there is a significant increase in IL-6 levels compared to partial seizures. Additionally, the IL-6 level is higher in chronic seizures than intermittent ones [27,28,29]. In animal models, TNF-α is quickly expressed during seizures. TNF-α has both proconvulsant and anticonvulsant effects, depending on its concentration in the brain (such as other cytokines) and the predominant receptor subtype activated in sick tissue. Recombinant mouse TNF-α inserted in mouse hippocampus resulted in a reduction in seizure activity by stimulating TNFR2, whereas it stimulated seizures by activation of TNFR1. Furthermore, a protective role of TNF-α is reported in mice with a genomic deficit of TNFR1. Additionally, signs of neurologic impairment involving seizures were elevated in mice with overexpression of TNF-α, while in transgenic mice with TNF-α at low-moderate levels, a decrease in vulnerability to seizures was reported [31].

The aim of neuroprotection is to prevent neuronal network and function [32]. Excessive and sudden stimulation of extra-synaptic NMDA receptors is neurotoxic [32]. Hence, in lieu of existing literature, it was inferred that combinations of CBZ and IMI would inhibit signaling through the PI3K/Akt/mTOR pathway, manifesting as a reduction in neuronal firing, alleviation of neuroinflammation, and prevention of neuronal network and function reorganization (i.e., neuroprotection). The neuroprotection would in turn preserve the structural and functional properties of receptors (which are otherwise altered by neurodegeneration, leading to a decrease in the responsiveness to drugs) and intercept the transformation of the brain from responsive to nonresponsive. Furthermore, the said combination could be used in refractory patients of epilepsy and those with epilepsy comorbid with depression. 

## 2. Results

### 2.1. Effects of Carbamazepine, Imipramine and Their Low Dose Combination on MES Induced Tonic Hind Limb Extension (THLE)

**Figure 1:**
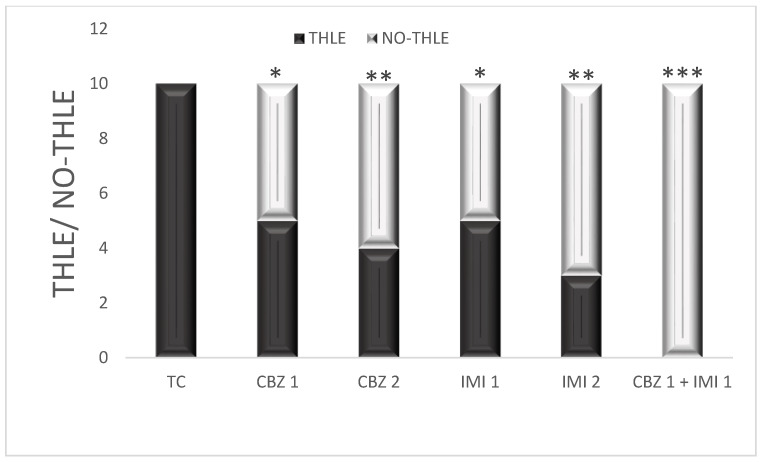
The effect of CBZ (20 and 50 mg/kg), IMI (10 and 20 mg/kg) and CBZ (20 mg/kg) + IMI (10 mg/kg) on MES-induced THLE. Statistical significance between toxic control and other groups was computed using Fisher’s exact test (one tailed). (*p* < 0.05 *, *p* < 0.01 **, *p* < 0.001 *** significance levels). CBZ, IMI, and TC indicate carbamazepine, imipramine, and toxic control, respectively.

In the toxic control group, the MES (180 mA, 220 V, 0.2s) escalated seizure activity to tonic hind limb extension in all rats (100% THLE). The CBZ dosed at 20 and 50 mg/kg abolished electroshock induced THLE (50% THLE, 40% THLE; *p* < 0.05, *p* < 0.01) respectively. The IMI dosed at 10 and 20 mg/kg also abolished the electroshock inculcated THLE (50% THLE, 30% THLE; *p* < 0.05, *p* < 0.01), respectively. The higher doses of CBZ and IMI were more protective than the lower doses (as monotherapies). Howbeit in combination, CBZ (20 mg/kg) and IMI (10 mg/kg) abolished THLE in all rats (0% THLE; *p* < 0.001), i.e., 100% protection against electroshock induced THLE. The efficacy of the combination therapy was thus far better than monotherapy with individual drugs. The pretreatment with all drugs was scheduled for 14 days. Statistical comparison of all groups with toxic control (Figure 1). 

### 2.2. Effects of Carbamazepine, Imipramine and Their Low Dose Combination on the Duration of Clonic Convulsions

**Figure 2:**
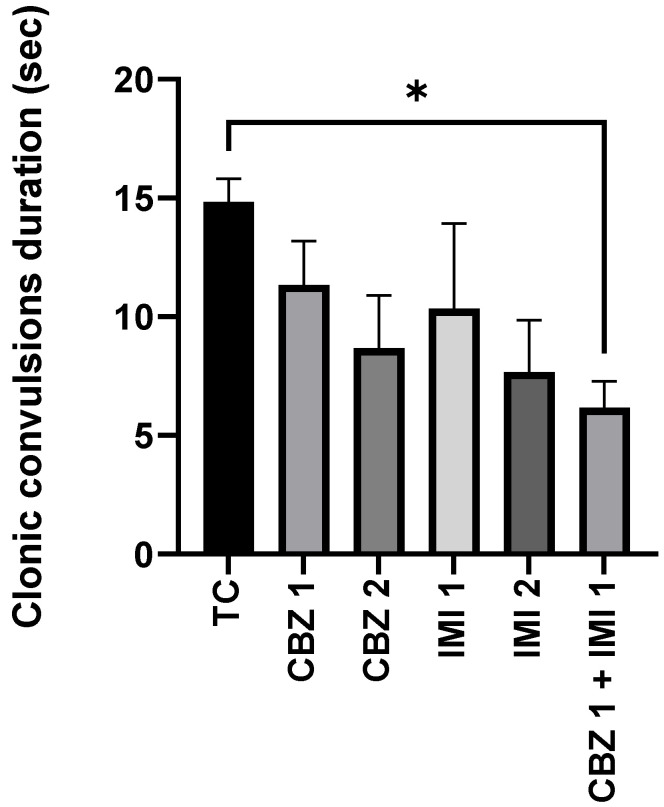
The effect of CBZ (20 and 50 mg/kg), IMI (10 and 20 mg/kg) and CBZ (20 mg/kg) + IMI (10 mg/kg) on the duration of clonic convulsions. Statistical significance between means from toxic control and other groups was correlated by applying one-way ANOVA followed by post hoc Dunnet’s test. *p* < 0.05 * was considered significant. CBZ, IMI, and TC indicate carbamazepine, imipramine, and toxic control, respectively.

Our data suggest that pretreatment with CBZ (20 and 50 mg/kg) and IMI (10 and 20 mg/kg) alone failed to reduce (significantly) the duration of clonic convulsions from the electroshock, relative to the toxic control group. Only the pretreatment with low dose combination therapy, i.e., CBZ (20 mg/kg) and IMI (10 mg/kg) significantly (*p* < 0.05) reduced the duration of clonic convulsions. Statistical comparison of all groups with toxic control (Figure 2).

### 2.3. Effects of Carbamazepine, Imipramine and Their Low Dose Combination on Pro-Inflammatory Makers

#### 2.3.1. Effect on Hippocampal IL-1β Levels 

**Figure 3:**
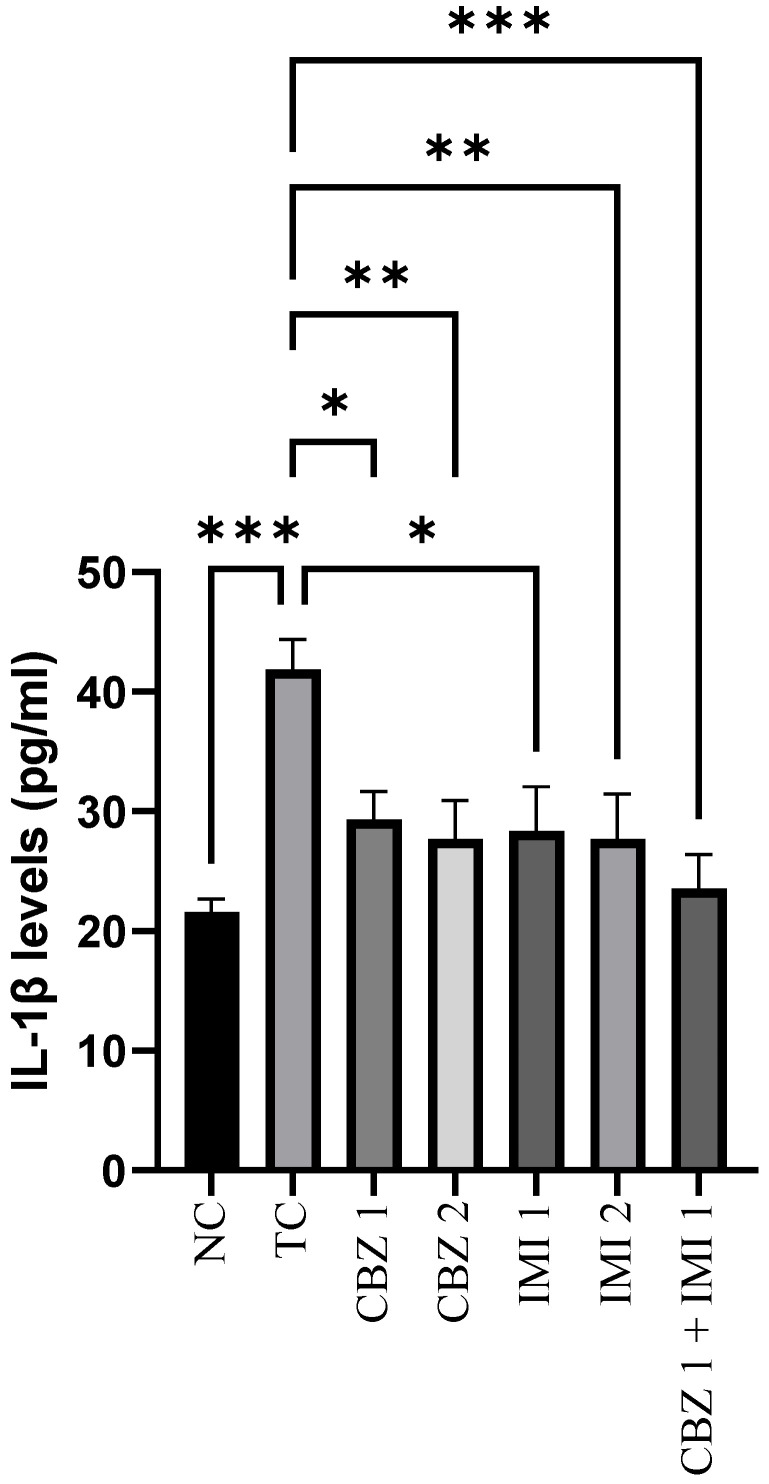
The effect of CBZ (20 and 50 mg/kg), IMI (10 and 20 mg/kg) and CBZ (20 mg/kg) + IMI (10 mg/kg) on hippocampal IL-1β levels. Statistical significance between means from toxic control and other groups was correlated by applying one-way ANOVA followed by post hoc Dunnet’s test. (*p* < 0.05 *, *p* < 0.01 **, *p* < 0.001 *** significance levels). CBZ, IMI, and TC indicate carbamazepine, imipramine, and toxic control, respectively.

Figure 3 shows the levels of IL-1β in the control and treated groups. MES escalated (*p* < 0.001) the levels of hippocampal IL-1β in the toxic control, in relation to the normal control group. However, pretreatment with CBZ at 20 and 50 mg/kg reduced (*p* < 0.05, *p* < 0.01) the IL-1β levels in comparison to the toxic control. Furthermore, pretreatment with 10 and 20 mg/kg doses of IMI abetted (*p* < 0.05, *p* < 0.01) the rise in IL-1β levels. Howbeit, the most significant effect (*p* < 0.001) was inculcated with the combination therapy entailing pretreatment with CBZ (20 mg/kg) and IMI (10 mg/kg). Statistical comparison of all groups with toxic control.

#### 2.3.2. Effect on Hippocampal IL-6 Levels

**Figure 4:**
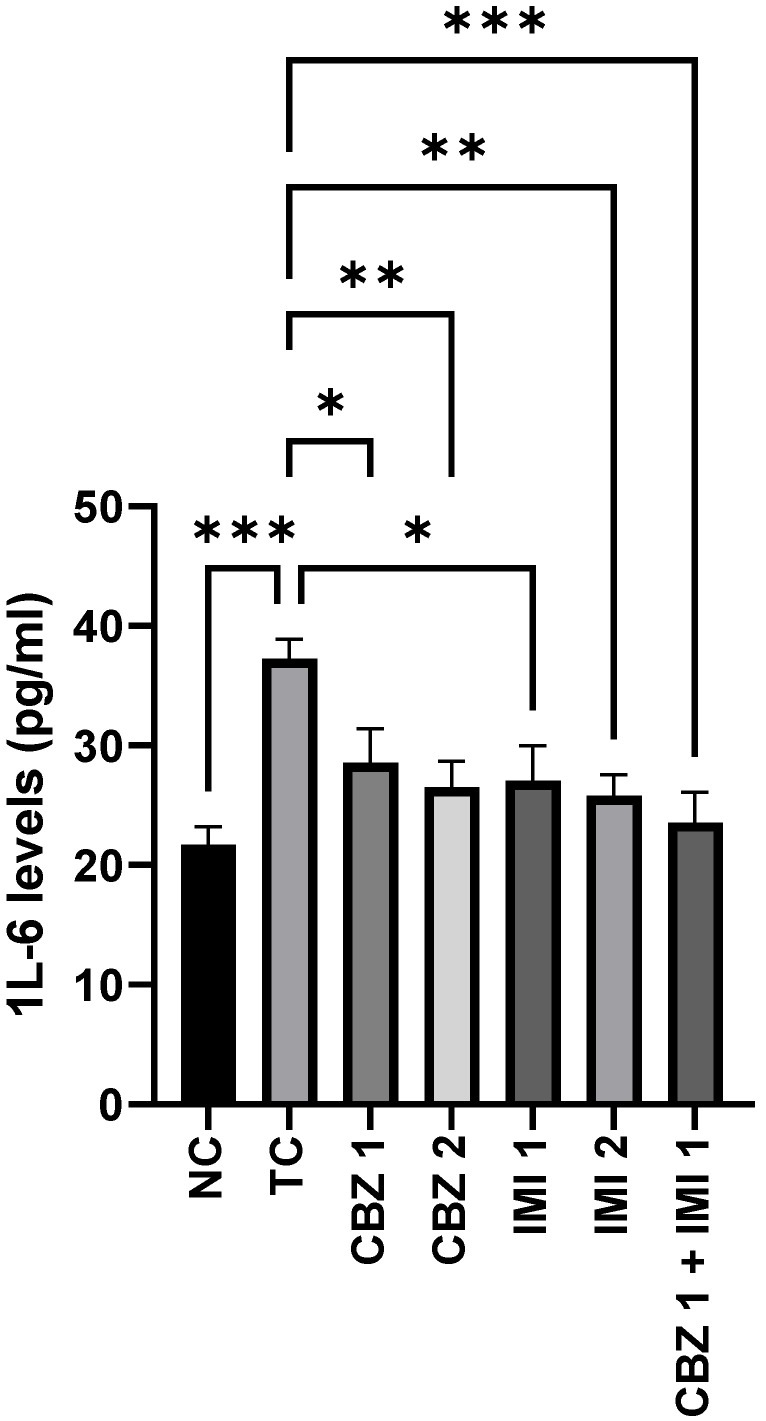
The effect of CBZ (20 and 50 mg/kg), IMI (10 and 20 mg/kg) and CBZ (20 mg/kg) + IMI (10 mg/kg) on hippocampal IL-6 levels. Statistical significance between means from toxic control and other groups was correlated by applying one-way ANOVA followed by post hoc Dunnet’s test. (*p* < 0.05 *, *p* < 0.01 **, *p* < 0.001 *** significance levels). CBZ, IMI, and TC indicate carbamazepine, imipramine, and toxic control, respectively.

Figure 4 shows the levels of IL-6 in the control and treated groups. MES escalated (*p* < 0.001) the levels of hippocampal IL-6 in the toxic control group, in relation to the normal control group. However, pretreatment with 20 and 50 mg/kg doses of CBZ alleviated (*p* < 0.05, *p* < 0.01) the IL-6 levels in relation to the toxic control. Furthermore, pretreatment with 10 and 20 mg/kg doses of IMI abetted (*p* < 0.05, *p* < 0.01) the rise in IL-6 levels. Howbeit, the most significant effect (*p* < 0.001) was inculcated with the combination therapy involving prior treatment with CBZ (20 mg/kg) and IMI (10 mg/kg). Statistical comparison of all groups with the toxic control.

#### 2.3.3. Effect on Hippocampal TNF-α Levels

**Figure 5:**
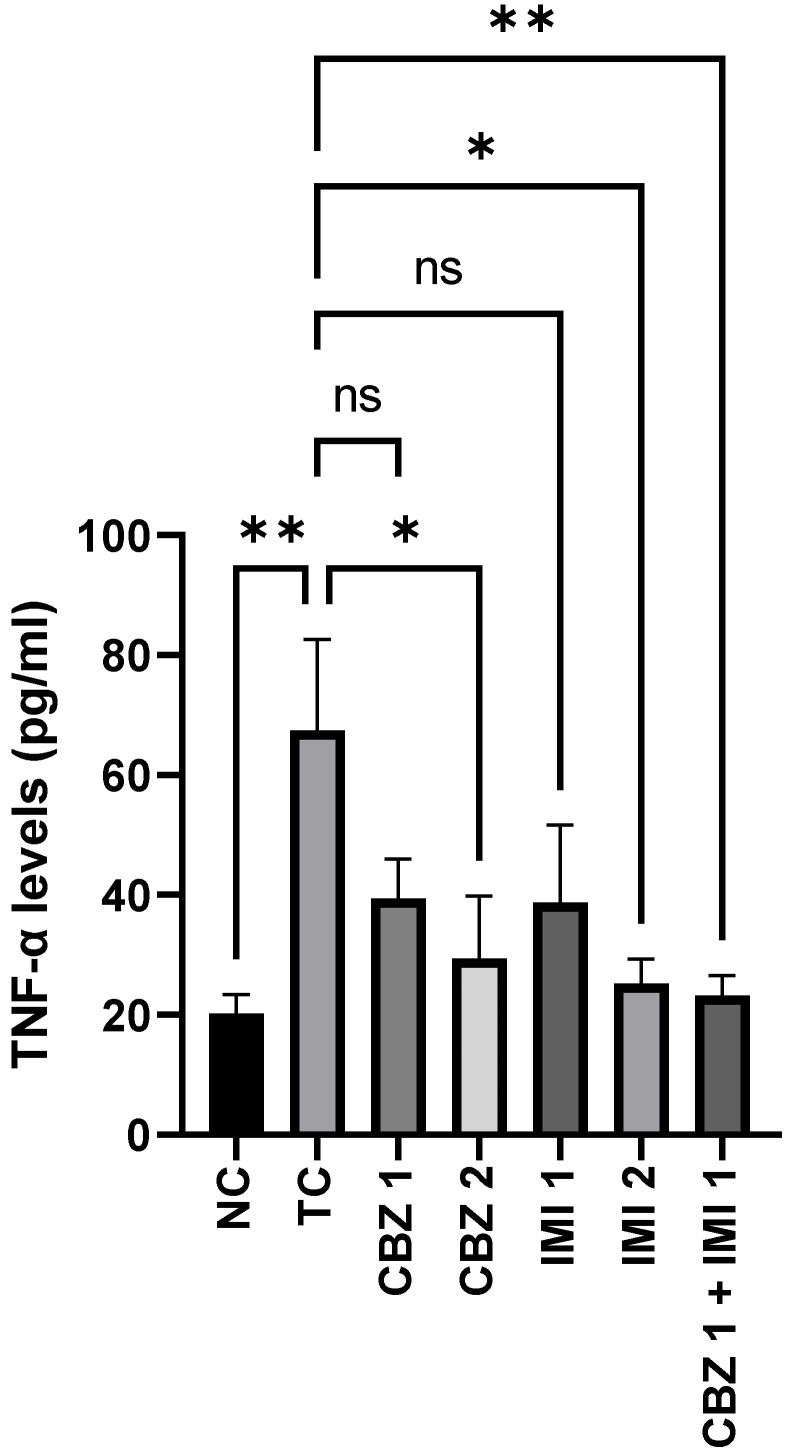
The effect of CBZ (20 and 50 mg/kg), IMI (10 and 20 mg/kg) and CBZ (20 mg/kg) + IMI (10 mg/kg) on hippocampal TNF-α levels. Statistical significance between means from toxic control and other groups was correlated by applying one-way ANOVA followed by post hoc Dunnet’s test. (*p* < 0.05 *, *p* < 0.01 ** significance levels). CBZ, IMI, and TC indicate carbamazepine, imipramine, and toxic control, respectively.

Figure 5 shows the levels of TNF-α in the control and treated groups. MES escalated (*p* < 0.01) the levels of hippocampal TNF-α in the toxic control group, in relation to the normal control group. However, pretreatment with lower doses, i.e., 20 mg/kg of CBZ and 10 mg/kg of IMI failed to significantly reduce the TNF-α levels in relation to the toxic control. While the corresponding higher doses, i.e., 50 mg/kg of CBZ and 20 mg/kg of IMI abetted (*p* < 0.05) the rise in TNF-α levels. Howbeit, the most prominent effect (*p* < 0.01) was inflicted with the combination therapy involving pretreatment with lower doses of CBZ (20 mg/kg) and IMI (10 mg/kg). Statistical comparison of all groups with the toxic control.

### 2.4. Effects of Carbamazepine, Imipramine and Their Low Dose Combination on the Hippocampal mTOR Levels

**Figure 6:**
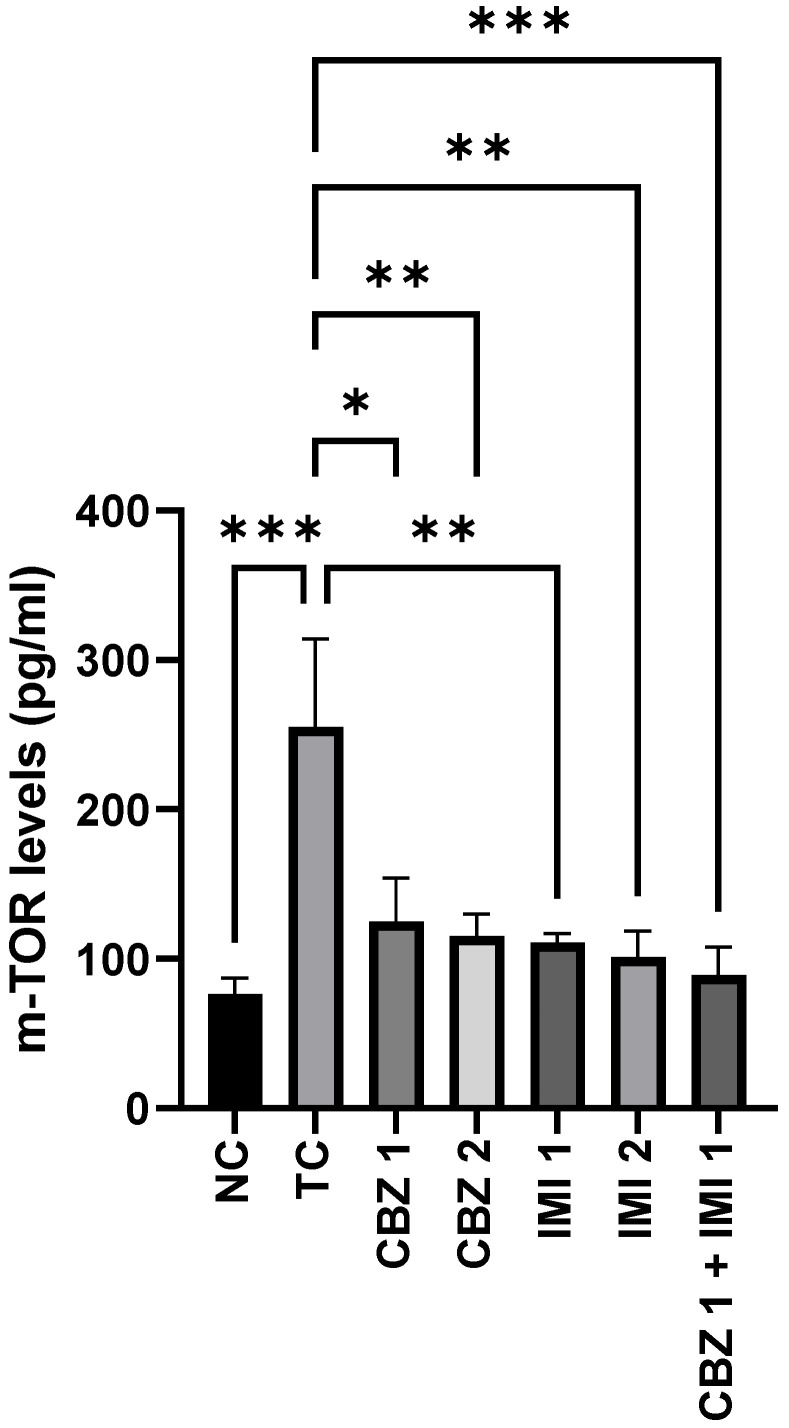
The effect of CBZ (20 and 50 mg/kg), IMI (10 and 20 mg/kg) and CBZ (20 mg/kg) + IMI (10 mg/kg) on hippocampal mTOR levels. Statistical significance between means from toxic control and other groups was correlated by applying one-way ANOVA followed by post hoc Dunnet’s test. (*p* < 0.05 *, *p* < 0.01 **, *p* < 0.001 *** significance levels). CBZ, IMI, and TC indicate carbamazepine, imipramine, and toxic control, respectively.

Our data indicate the significant role of the mTOR signaling pathway in epileptic seizures, as electroshock instilled a significant (*p* < 0.001) rise of hippocampal mTOR levels in the toxic control group, in relation to the normal control. Pretreatment with 20 and 50 mg/kg doses of CBZ reduced (*p* < 0.05, *p* < 0.01) mTOR levels. Furthermore, pretreatment with 10 and 20 mg/kg doses of IMI reduced (*p* < 0.01) mTOR levels, but the effect was the same at both dose levels. Interestingly, the combinatorial therapy exhibited maximal effect (*p* < 0.001) in halting the invigoration of the mTOR pathway, which reveals the potential of the said combination to avert brain transformation from normal to epileptic. Statistical comparison of all groups with the toxic control (Figure 6). 

### 2.5. Effect of Carbamazepine (CBZ), Imipramine (IMI) and Their Low Dose Combination on the Hippocampal Neuronal Damage

**Figure 7:**
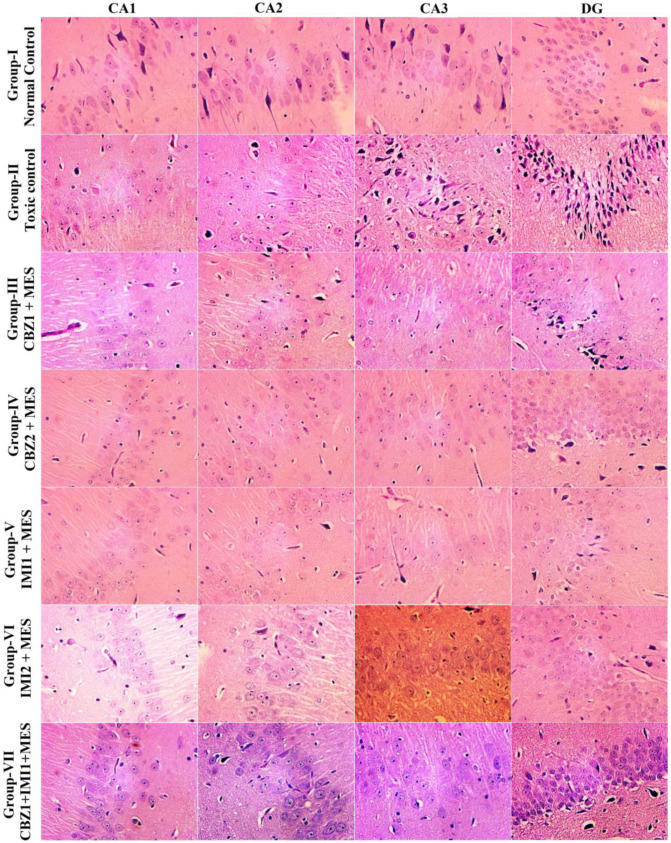
H/E stained rat hippocampal sections from normal control (group-I), toxic control (group-II) and drug-treated (groups III–VII). The differential effects of treatment with CBZ (20 and 50 mg/kg), IMI (10 and 20 mg/kg) and CBZ (20 mg/kg) + IMI (10 mg/kg) on the hippocampal neurons after electroshock are shown. It seems that the higher doses of CBZ and IMI are more protective against deteriorating effects of electric shock (normal neuronal density is more in those groups), compared to their lower doses. Howbeit, the combination therapy seemed to largely restrict the damage in CA1, CA2 and DG areas, while the neuronal density of the CA3 region seems to be reduced. Clearly the neuroprotective effects of the combination group exceed other treated groups.

The dynamics of neurodegeneration from seizures and the effect of drugs were probed from histomorphological studies. The electroshock in the toxic control group culminated in heavy neuronal reduction in all hippocampal regions CA1, CA2, CA3 and DG regions, while pyramidal layer neuronal atrophy (stained dark by H&E) was observed in CA3 and DG areas. Pretreatment with test drugs; CBZ (20 and 50 mg/kg) and IMI (10 and 20 mg/kg) raised the threshold for electroshock insults and thereby restricted neuronal loss and pyknosis, as evidenced from the photomicrographs. The higher doses were more protective than the lower doses. Howbeit, the combination of 20 mg/kg CBZ and 10 mg/kg IMI unfolded significant neuroprotection, given that the neuronal loss and pyknosis seem to be highly restricted (Figure 7). 

### 2.6. Cell Viability Assay

**Figure 8:**
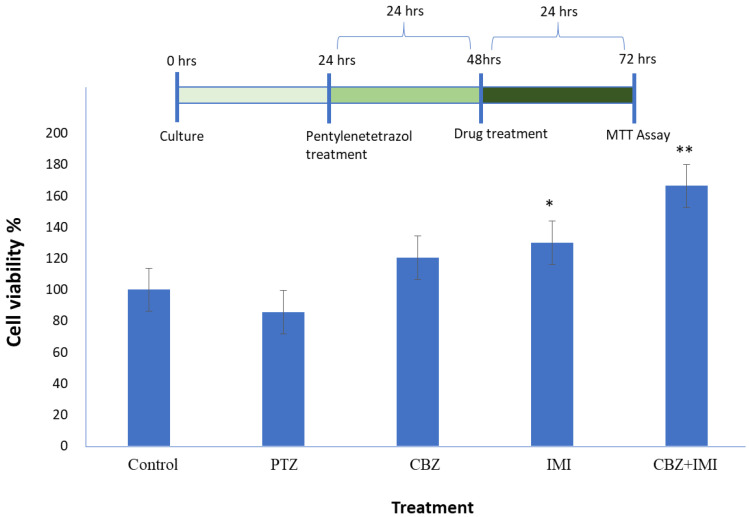
Cell viability assay by MTT: HEK-293 cells treated with vehicle (Control group), PTZ (0.6 µg/mL), CBZ (0.35 µg/mL), IMI (0.35 µg/mL), CBZ + IMI (0.70 µg/mL + 0.35 µg/mL). The cells were first treated with PTZ for 24 h, then exposed with CBZ, IMI, CBZ + IMI for 24 h. The % of cell viability given in the graph is taken from the dose that gave the highest percentage of cell viability. The cells were exposed with doses ranging from 0.2 to 0.90 µg/mL. The difference between the control and drug-treated groups was computed using ANOVA, *p*-values were computed by Student’s *t*-test; *p* < 0.05 *, *p* < 0.01 ** significance levels.

Cell viability was assessed by MTT assay. The HEK-293 cells exhibited 100% cell viability (normal control). However, the cell viability was reduced to 85.66% in the toxic control group, which was exposed to PTZ (0.6 µg/mL) for 24 h. The PTZ treated cells were further exposed to test drugs, CBZ and IMI alone, and in combination for 24 h. The treatment with CBZ (0.35 µg/mL) and IMI (0.35 µg/mL) resulted in upsurge of cell viability to 120.37% and 130% with respect to PTZ-treated cells (85.66%). Interestingly, the treatment of cells with the combination (CBZ 0.70 µg/mL + IMI 0.35 µg/mL) fetched the largest increase in cell viability (166.37%), which indicates that the combination therapy with CBZ and IMI is most protective against the damaging effects of PTZ (Figure 8). 

### 2.7. Molecular Docking

CBZ and IMI were passed through molecular docking simulation for their cooperative binding capability to Akt (PDB code; 4gv1). Each compound was first screened to whether it preferably binds to allosteric or orthosteric pocket and binding scores were calculated for the individual compounds. Allosteric and orthosteric key binding residues were identified from reference crystal structures. The results showed that CBZ preferably binds the allosteric site with a binding affinity of −7.8, while IMI binds the active site with a binding affinity of −8.4. The second step was to dock the second drug in the presence of the first drug in the corresponding pocket, two docking experiments were performed starting with the enzyme with the first docked compound in the corresponding pocket followed by docking the second drug, and binding scores were calculated for the new enzyme drugs complex after docking. In both cases better results were observed when CBZ occupied the allosteric pocket first with a binding score of −12.5 compared to when IMI occupied the binding site first with a binding score of −8.6 (Table 1). These results actually supports the proposed mode of binding where the allosteric modulator potentiates the action of another molecule that binds the active site. Comparative docking was also performed by comparing the ligand positions in the co-bound form with the reference crystallized inhibitor (AZD5363).

**Figure 9:**
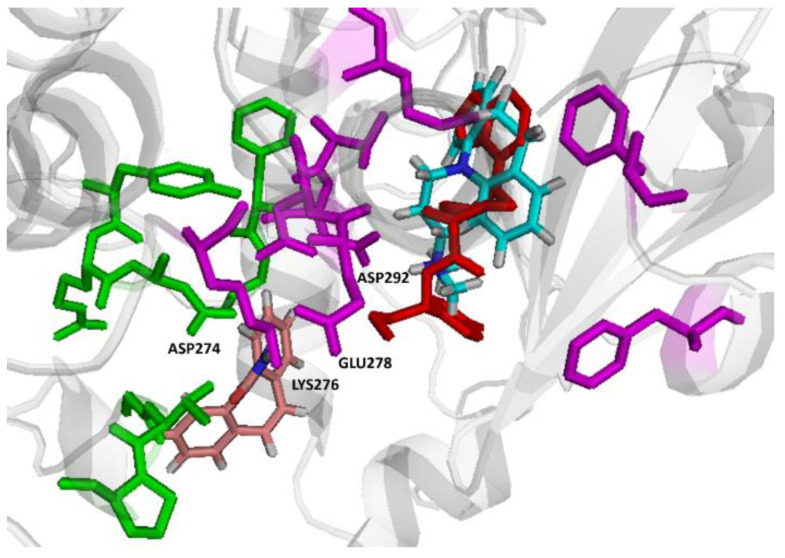
Comparative binding positions of co-bound CBZ (brown) bound to an allosteric pocket (residues shown in green) and IMI (light blue) with the reference crystallized ligand, AZD5363 (red) bound to Akt (PDB Code: 4GV1) active site (residues shown in magenta).

**Figure 10:**
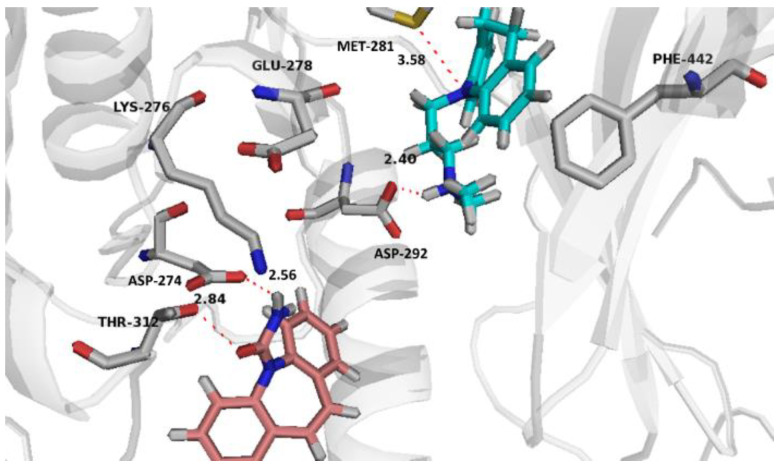
Binding interactions of co-bound CBZ (carbons in brown) and IMI (carbons in light blue) with the Akt (PDB Code: 4GV1) allosteric site and active site, respectively. Distances are represented as red dotted lines and are measured in Angstrom (Å).

Both drugs were found to cooperatively bind the orthosteric and allosteric sites of Akt, and interesting binding poses were noted where CBZ binds the allosteric site, while IMI almost superposes the reference crystallized ligand in the active site (Figure 9). CBZ and IMI were found to cooperatively bind the allosteric site and the active site of Akt, respectively, and establish a good network of intermolecular interactions (Figure 10). CBZ established a strong hydrogen bond with its amide side chain nitrogen and the key allosteric residue ASP-274 sidechain carboxylate (the bond length is 2.84 Å, which is considered strong). Another hydrogen bond was also noted to the amide side chain oxygen and THR-312. IMI binds the active site mainly through a strong hydrogen bond with its side chain secondary nitrogen and the key residue ASP-292 (the bond length is 2.40 Å, which is considered strong).). A potential hydrogen bond was also noted between the ring nitrogen and MET-281 sidechain. The initial binding of CBZ to the allosteric site potentiates the binding of IMI to the active site potentially through decreasing the interaction between ASP-274 and LYS-276, which on the other hand, leads to promoting the interaction of LYS-276 alternatively with GLU-278, which possibly serves as a gate keeper. The net result is moving GLU-278 away from the active site and opening the way for IMI to enter the active site and take a position that allows optimal binding with the key residues ASP-292 and MET-281 (Figure 10). The cooperative binding of CBZ/IMI to Akt shown in silico could explain their synergistic action on weakening the mTOR signal.

## 3. Discussion

Even though all epilepsy patients have seizures, but not all seizures are considered epilepsy [6]. Seizures can occur secondary to acute CNS triggers such as metabolic condition, drug toxicity and CNS infections [33]. Seizures are of two major types (a) focal onset, seizures originate from one hemisphere of the brain; (b) generalized seizures, which originate from both brain hemispheres simultaneously. Status epilepticus (SE) is a prolonged seizure condition, which occurs repeatedly at transient intervals, causes long-term consequences, and can lead to death [6]. The prognosis of seizure is favorable and good if the patient responds to medication and is measured as seizure-free [6]. According to Sander, epileptic patient prognosis is classified into four groups: (a) About 20% to 30% of total patients have a good prognosis with benign myoclonic and benign focal epilepsies in infancy; (b) good prognosis in about 30% to 40% of patients with easy pharmacological control, including absence seizures of childhood and focal type; (c) undefined prognosis in about 10–20% of patients, whom respond to anti- epileptic drugs (AEDs) but have seizure recurrence after treatment cessation [6]; (d) poor prognosis in about 20%, which means seizures occur despite the intensive treatment, including epilepsies associated with congenital impairment, progressive neurological condition, and some cryptogenic partial epilepsies [6]. The actual drug for epilepsy was discovered in 1912 when phenobarbital was introduced as an effective anti-epileptic drug (AED) [34]. Eventually more AEDs were introduced to the market; phenytoin, benzodiazepines, ethosuximide, valproate, and carbamazepine categorized as first generation. Vigabatrin, pregabalin, tiagabine, gabapentin, lamotrigine, oxcarbazepine, topiramate and levetiracetam as second generation [35]. In the latest AEDs, the third generation includes lacosamide, perampanel, eslicarbazepine and brivaracetam [34]. In clinical practice, clinicians start with monotherapy in newly diagnosed patients, and depending upon the patient response, a combination of medication is used to attain the therapeutic goal [6,35]. As the AEDs usage has increased in the past decade, the number of combination regimens has also multiplied [34]. The most common anti-epileptic combination regimens are: lamotrigine/topiramate for various seizure types, phenobarbital/phenytoin for generalized “grandmal” seizure and carbamazepine/Valproic acid for partial seizures [36]. Despite the use of these combinations, 35% of patients still remain refractory, and toxicity associated with these combinations cannot be ignored [37]. Therefore, we followed a progressive preclinical investigation in rats to test whether or not IMI (antidepressant) would potentiate the anticonvulsant efficacy of CBZ (as two drugs have different modes of actions). The experimental results revealed that the low dose combination of CBZ and IMI exhibited a synergistic anticonvulsant effect and that the combination inhibits neuronal inflammation by reducing pro-inflammatory cytokine levels and intercepts mTOR signaling. In silico studies confirmed the synergistic action shown by the CBZ + IMI on weakening the upstream signal of mTOR namely Akt (both drugs were also found to cooperatively bind the orthosteric and allosteric sites of Akt). In addition, the said combination when tested on HEK-293 cells increased cell viability by 176.72% compared to PTZ (neurotoxin)-treated HEK-293 cells, i.e., the combination is neuroprotective as well. 

CBZ is a known AED, which works by the blockade of voltage-dependent Na^+^ channels in two ways: (a) inhibition of Na^+^ channels in the resting state; and (b) the blockade of Na^+^ channels in use-dependent mode [38]. CBZ reduced the motor seizure rate in rats with kainite induced epilepsy [39]. CBZ produced a significant reduction in convulsions produced via tetanus toxin injected bilaterally to the rat hippocampus (EEG revealed decrease in seizure discharge) [40]. A study linked the anticonvulsant activity of CBZ with cholinergic receptor inhibition [41]. However, the use of CBZ is sometimes limited due to serious adverse effects, such as aplastic anemia and agranulocytosis. Furthermore, the pregnancy category is D, so clinicians use it if the benefits outweigh the increased risk of congenital malformations such as spina bifida and developmental delays [42]. IMI antagonizes alpha 1/2 adrenergic receptors and Histamine (H1) receptors [43,44]. IMI has been reported in some studies as a possible treatment for epilepsy. Investigators had reported its effect on myoclonic astatic type, generalized absence, and temporal lobe epilepsy. The mechanism is still unrevealing, and some studies suggest IMI might work like ethosuximide [43,44], such as the inhibition of corticofugal in the trigeminal nucleus and then, eventually, the prevention of seizure activity spreading throughout the subcortical area. In an in vitro study, segments of the hippocampus isolated from Wistar rats were dipped in different anticonvulsant solutions [43,44], and IMI reduced the convulsion-like effect (SLE) gradually till total irreversible suppression of seizure movement in all segments [43]. Much to the contrary, some studies revealed dual action of IMI on the CNS, i.e., the anti-seizure effect at small doses and pro-convulsant effects at higher doses [43,44]. In animals challenged with maximal electroshock (MES), IMI (17.5 and 25 mg per kg) inhibited seizure activity. However, in PTZ-induced seizures, there was no effect [45]. In agreement with most studies, we found that CBZ and IMI exhibit dose-dependent anticonvulsant effects, but the low dose combination therapy of CBZ (20 mg/kg) + IMI (10 mg/kg) exhibits synergism (*p* < 0.001) in abrogation of electroshock induced seizures in rats. 

Large studies in the past two decades have proven a central role for mTOR in the regulation of essential cell function, ranging from protein synthesis to autophagy [22]. On the other hand, dysregulation of mTOR signaling is linked to cancer, diabetes and the aging process [22,46]. mTOR plays a role in memory and neuronal elasticity, and it is reasonable that mTOR activity is related to the pathophysiology of several CNS conditions, such as Huntington’s disorder, bipolar disorder and depression. Therefore, targeting the mTOR pathway might be beneficial to understanding the pathophysiology of such disorders and for the discovery of novel remedial approaches [23]. Furthermore, the PI3K/Akt/mTOR route exists extensively in neurons and controls the biological roles of nerve cell proliferation, metabolism, differentiation and apoptosis [47]. The PI3K/Akt/mTOR pathway exhibits a significant role in neurodegenerative diseases such as epilepsy and Parkinson’s disease. miRNA-155 provokes the beginning of convulsions in epilepsy via the PI3K/Akt/mTOR pathway [48]. CBZ inhibited lipopolysaccharide-activated phospho-Akt expression in microglial BV-2 cells [49]. Park et al., 2014, reported that IMI had no effect on mTOR levels in rat hippocampus [50]. However, other studies report that IMI inhibited signaling through PI3K/Akt/mTOR in U-87MG human glioma cells [51]. In line with the existing literature, we found hyperactivation of the mTOR pathway upon electroshock induced seizures, while CBZ and IMI reduced the activation; however, the most significant (*p* < 0.001) suppression of the mTOR pathway was evidenced from the low dose combination therapy, i.e., CBZ (20 mg/kg) + IMI (10 mg/kg). This indicated that the said combination inhibits upstream signals of the mTOR pathway as well, which was confirmed from in silico studies, revealing the inhibition of Akt by this combination therapy. The insilico (computational) studies also uncovered cooperative binding between CBZ and IMI at Akt, thus the two drugs in combination potentiate their binding to the target and hence the efficacies, along with lowering of toxicities, as low doses were combined. 

Inflammatory signaling, such as stimulating cytokine release and related immunological process, similarly show an essential part in the persistent seizure [24]. Recent studies suggest that pro-inflammatory cytokines, including IL-6, IL-8, IFN-γ, are possible pro-convulsant cytokines. IL-1 systemic cascade in febrile conditions promotes the entry of peripheral cytokines into the CNS and lowers the seizure threshold. However, at the same time, the febrile conditions show elevation of anti-inflammatory cytokines such as IL-10 and IL-1Ra, as a compensatory mechanism [52]. The inhibition of pro-inflammatory pathways may prevent the development of chronic seizures [24]. CBZ and vinpocetine reduced the expression of tumor necrosis factor and IL-1b from basal states induced by LPS in the rat hippocampus [53]. IMI reduced the levels of TNF-α and IL-1β in primary rat mixed glial cell culture stimulated by LPS [54]. IMI reduced IL-6 in plasma of 57BL/6 mice exposed to stress [55]. IMI reduced the levels of IFN-γ, IL-6 in the rat hypothalamus [56]. In line with the previous studies, we found that the drugs, CBZ and IMI, alleviate the neuronal hyperinflammation by reducing the levels of pro-inflammatory cytokines (IL-1β, IL-6 and TNF-α). Howbeit, the most significant (*p* < 0.001) anti-inflammatory property was exhibited by the low dose combination therapy, i.e., CBZ (20 mg/kg) + IMI (10 mg/kg).

Seizure inculcated neurodegeneration triggers various pathological responses such as inflammatory signaling, synaptic plasticity and migration of surviving neurons and glial cells [57]. In addition to anti-seizure activity, carbamazepine (CBZ), valproic acid (VPA), and topiramate (TPM) demonstrated neuroprotection in an in vitro ischemia model, in part due to the inhibition of fast Na^+^ and HVA Ca^2+^ conduction [58]. Oxcarbazepine protected neuronal cells from damage in the gerbil hippocampus induced by transient global cerebral ischemia and drastically reduced glial cell activation in the ischemic hippocampus [59]. Our study revealed that the higher dose of CBZ and IMI exhibit protection from damaging effects of electroshock, while the best effect was evidenced with a low dose combination therapy, i.e., CBZ (20 mg/kg) + IMI (10 mg/kg), as the extent of neuronal damage was limited compared to other treated groups. 

## 4. Materials and Methods

### 4.1. Animals 

All animals were obtained from the Animal House, IRMC, IAU, Dammam. We utilized Wistar rats (Female and male; 8–10 weeks old in the weight range of 180–240 g). The rats were kept in standard cages below natural light on/off cycles and specific humidity (55–65%) and temperature (25 ± 2 °C). They were on a normal diet. The day prior to the experiment, the rats were adapted to the laboratory environment. The approval for this study was received from IACUC, IAU (Approval no: IRB-2021-05-303). 

### 4.2. Drugs and Dosing Schedule

The investigated drugs were: carbamazepine (CBZ; 20 and 50 mg per kg), imipramine (IMI; 10 and 20 mg per kg). All drugs were dispensed in 2% Tween 20. Oral route was used for drug administration, which continued for 14 days prior to the MES challenge. 

### 4.3. Experimental Groups 

Seventy rats were randomized into 7 groups (10 rats/group). Groups I and II were given 0.2 mL of 2% Tween 20 (p.o). Group III–IV were given CBZ at 20 and 50 mg/kg (p.o). Group V–VI were given IMI at 10 and 20 mg/kg (p.o). Group VII was given a combination of CBZ (20 mg/kg, p.o) and IMI (10 mg/kg, p.o). Seizures were induced by electroshock apparatus to all groups on the last day of dosing, except normal control (Group-1).

### 4.4. MES induced THLE 

Maximal electroshock seizure (MES) is a preclinical investigational model that produces synchronal neuronal discharges in the brain through synthetic current input to mimic acute epileptic states [60]. We applied an alternating current (180 mA, 220 V for 0.20 s) supplied by a generator through electrodes pined in the ear. The characteristic behaviors of rats following electric shock were recorded into the following stages; phase-1: tonic limb flexion, phase-2: tonic limb extension, phase-3: clonic convulsions, phase-4: stupor and phase-5: recovery or death [61]. The behavioral monitoring of convulsions continued for 0–30 s, during which different stages of convulsions exhibited by the rats were recorded until the animal gained the posture and/or started moving normally. 

### 4.5. Experimental Design 

The rat group’s I-II received vehicle, and groups III-VII received drug regimen (CBZ1, CBZ2, IMI1, IMI2, CBZ1 + IMI1) for 14 days. All rats (10/group) from groups II-VII were challenged with Maximal Electroshock (180 mA, 220 V for 0.20 s) on the last day of the treatment schedule. Thereafter, six rats from each group were sacrificed by the use of sevoflurane for the removal of brain, which was further dissected for the extraction of the hippocampus. The hippocampus was preserved at −80 °C, and was later processed for estimation of mTOR, IL-1β, IL-6 and TNF-α using ELISA technique. The four rats left from each group were surveilled for mortality for 1 day, and thereafter sacrificed for excision of the brain, which was stored in formalin 10%, and later used for histopathological investigations. 

### 4.6. MES Induced Neuronal Damage 

One day after the MES challenge, histopathological assessment of the rat hippocampus was conducted with the aim of accessing the cellular changes in hippocampal regions; CA1, CA2, CA3 and DG. The brains were sliced coronally (thickness 8-μm) using the microtome at 2.3 to 4.3 mm bregma to the posterior area. Then slices were stowed on glass slides and stained with H/E (hematoxylin and eosin). The pictures were taken from hippocampal areas; DG, CA1, CA2 and CA3 by a pathologist using a light microscope (40× magnification). 

### 4.7. Assessment of Hippocampal mTOR Levels

The hippocampal supernatant was used for the quantification of mTOR levels by ELISA. The protocol from the manufacturer (Aviva Systems Biology; San Diego, CA, USA) was chased. 

### 4.8. Assessment of Inflammatory Markers in the Hippocampus

The TNF-α, IL-1β and IL-6 were quantified from the hippocampal supernatant by using the ELISA kit from Aviva Systems Biology (San Diego, CA, USA). The protocol from Manufacturer was chased. 

### 4.9. Invitro Studies

#### 4.9.1. Cell Culture

We utilized HEK-293 (human embryonic kidney cells) cultured in 96-well dishes containing DMEM media fortified with selenium chloride, streptomycin, fetal bovine serum, L-glutamine, and penicillin. Since we needed 70–80% confluence cells, these cells were kept in the (5%) CO_2_ incubator at 37 °C at least for 2–3 days. This method has been described previously by Pottoo et al., 2021 [61].

#### 4.9.2. Invitro Model of Cellular Degeneration

The cellular degeneration in the HEK-293 cells was instilled by exposure to Pentylenetetrazol (PTZ; 0.6 µg/mL) for 24 h. Afterwards, cellular anatomy and morphology were viewed under the light microscope. The PTZ is known to induce a generalized type of seizures in rats and is known to amplify signaling through mTOR [62,63]. 

#### 4.9.3. Treatment with Test Drugs

After induction of cellular degeneration in HEK-293 cells from 24 h of PTZ exposure, the cells were then exposed to the test drugs; CBZ (0.35 µg/mL), IMI (0.35 µg/mL), CBZ + IMI (0.70 µg/mL + 0.35 µg/mL) for 24 h. 

#### 4.9.4. MTT Assay

After completion of treatment, the HEK-293 cells were incubated with MTT (5.0 mg/mL) for 4 h. The cells were then rinsed, and 100 uL DMSO was poured into each well. The ELISA Plate Reader was used to determine the % cell viability at 570 nm wavelength (Biotek Instruments, Winooski, VT, USA). The detailed method has been described earlier [64,65]. 

### 4.10. Molecular Docking 

Molecular docking is a common computational procedure method used in structure-based drug design, it is an important tool to investigate the orientation between the ligand and receptor, intermolecular interactions and ligand binding which stabilizes the ligand-receptor complex. Molecular docking quantifies the binding energetics and ranks docked compounds based on binding affinity of ligand-receptor complexes [66]. Cooperative binding occurs when a ligand facilitates the binding of another ligand. Cooperativity might be negative (infralinear) or positive (supralinear). Mostly this type of binding is found in proteins, though nucleic acid also shows cooperative binding. Cooperative binding is the mechanism of many physiological and biochemical processes [67]. 

Molecular modeling software was used for molecular docking simulation and ligand binding energy calculation. Pymol was utilized for output data visualization and figure generation. The target employed was the crystal structure of human Akt (PDB code; 4gv1), co-crystallized with inhibitor (AZD5363). The Akt was used after deleting the co-crystallized inhibitor. All hydrogens were added to the ligand PDB file and partial charges were computed. The docking was performed using the MOE dock tool in MOE, with the default values. To explore the possible binding of ligands to allosteric sites, whole protein was used to define the active site. For simultaneous binding, the active site was defined by residues in the active site involved in binding the co-crystallized ligand. The docking results were evaluated using binding energy calculation and checking ligand binding position through interaction with key residues, and additionally validated through comparison with the crystallized ligand position.

### 4.11. Statistical Analysis 

The mean ± SEM of all groups was calculated. ANOVA followed by post hoc Dunnet’s was used to measure the level of significance between toxic control and other groups. The Fisher’s exact test (one tailed) was used for comparing the ratio of THLE/NO-THLE between toxic control and other groups. The *p*-value was set at *p* < 0.05 in all cases. 

## 5. Conclusions

Epilepsy causes social, economic, psychological, neurological and cognitive consequences. It affects the individual’s quality of life, impairs daily activity and might end in fatal conditions such as brain trauma, metabolic disorder, drug toxicities and stroke. The epileptic symptoms are presently controlled with AEDs and their combinations; however, treatment failure is observed in 35% of patients, which indicates that some additional mechanisms also contribute to epilepsy. We thus designed a prospective preclinical trial in rodents to combine anticonvulsant, CBZ, with tricyclic antidepressant, IMI. As the two drugs have different modes of actions, it was likely that synergistic effect from such combination would not only be highly efficacious but also reduce the toxicities of both drugs, and that such combination could also be used in epileptic patients with depression. The results eventually confirmed the synergistic potential of the combinatorial therapy; CBZ (20 mg/kg) + IMI (10 mg/kg). The said combination also reduced pro-inflammatory markers in the brain and intercepted signaling through the PI3K/Akt/mTOR pathway (which is otherwise hyperstimulated during seizures). These results were supported by in silico and in vitro studies. 

## Figures and Tables

**Table 1:** Dockings scores for carbamazepine, imipramine alone and co-bound in Akt.

Akt	**Docking Score**			
	**Carbamazepine 1st**	**Carbamazepine/Imipramine**	**Imipramine 1st**	**Imipramine/Carbamzepine**
	−7.8	−12.5	−8.4	−8.6

## Data Availability

All data are contained within the article.
